# Senile Calcification of the Trachea, Aortic Arch, and Mitral Annulus: An Incidental Finding on Chest X-Ray

**Published:** 2015-10-27

**Authors:** Mahmood Hosseinzadeh Maleki, Toba Kazemi, Navid Davoody

**Affiliations:** *Atherosclerosis and Coronary Artery Research Center, Birjand University of Medical Sciences, Birjand, Iran.*

**Keywords:** *Calcification*, *physiologic*, *Trachea*, *Aorta*, *Mitral valve*

## Abstract

A 94-year-old woman presented with dizziness and hypotension of 2 days’ duration. She denied any syncope, presyncope, or angina. She had received a permanent pacemaker 12 years previously for the management of complete heart block (CHB), but she failed to program it. Twelve-lead electrocardiography revealed CHB with ventricular escape rhythm (40/min), so we inserted a temporary pacemaker. Anteroposterior chest X-ray showed trachea, aortic arch, and severe mitral valve calcification.

Tracheal calcification is usually seen after 40 years old without clinical importance. However, it is seen in patients with renal failure, metastases, and prolonged use of warfarin as well as in pregnancy.^1^^-^^3^

**Figure 1:**
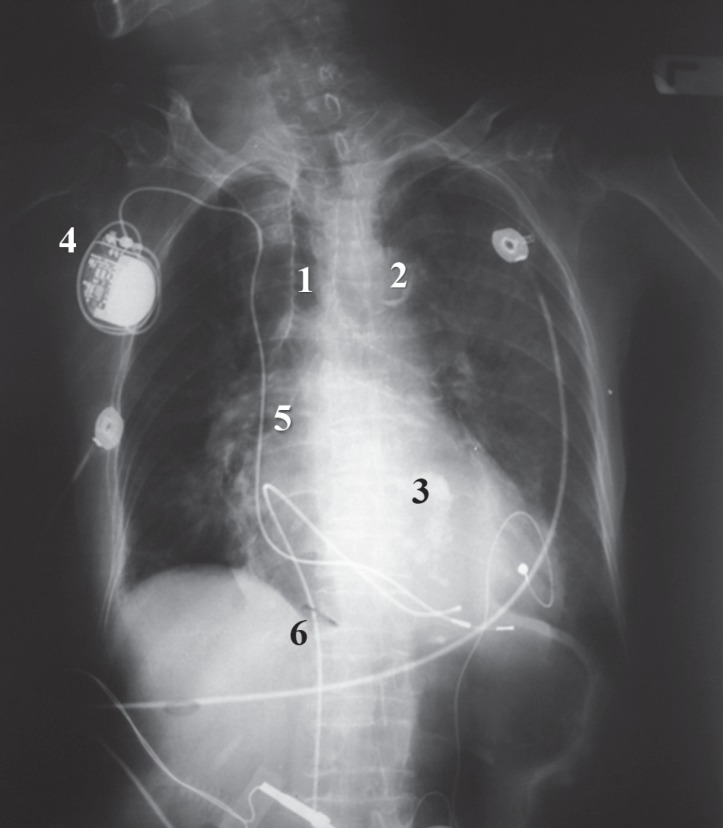
Chest X-ray shows calcification of the trachea (1), aortic arch (2), and mitral annulus (3). It also demonstrates the permanent pacemaker (4), and two leads from the permanent (5) and the temporary (6) pacemakers.

## Notes:

***This paper should be cited as:*** *Hosseinzadeh Maleki M, Kazemi T, Davoody N. Senile Calcification of Trachea, Aortic Arch and Mitral Annulus: An Incidental Finding on Chest X Ray. J Teh Univ Heart Ctr 2015;10(4):219-220.*
